# Supplementary material for: WordCommentsAnalyzer: A windows software tool for qualitative research
Source: F1000Res. 2018 Sep 4;7:536. Originally published 2018 May 3. [Version 2] doi: 10.12688/f1000research.14819.2 (PMC6137411; doi:10.12688/f1000research.14819.2)
Supplement: Supplementary file 3 [file f1000research-7-17614-s0002.tgz › b2a79496-4ada-47c4-9682-a99ce867836d.zip!/Eight Iranian Health Journals/Journal of Health System Research _16.docx]

© Journal of Health System Research . This work is licensed under CC BY-NC 4.0 (https://creativecommons.org/licenses/by-nc/4.0/).

Volume & Issue: دوره 10، شماره 2، 1393

Title: نقشه‌‌بندي تغييرات مكاني بيماري‌ها با استفاده از مد‌ل‌هاي كلاسيك و بيزي

http://hsr.mui.ac.ir/index.php/jhsr/article/view/1265

نقشه‌بندی بیماری شامل مجموعه‌ای از روش‌های آماری است که به تهیه نقشه‌های دقیق براساس برآوردهایی از میزان‌های بروز، شیوع و مرگ و میر منجر می‌شوند تا کاربران بتوانند با توجه به توزیع جغرافیایی بیماری‌ها، برنامه‌ريزي‌هاي بهداشتي لازم را با دقت بيشتري انجام دهند. اهداف اصلی نقشه‌بندی بیماری عبارت هستند از: تشریح تغییرات فضایی در شیوع بیماری به منظور فرمول‌بندی فرضیات علت‌شناسی، ارزیابی خطر و تعیین نواحی دارای خطر بالا که نیازمند جلب توجه و اعمال مداخله هستند، تهیه نقشه دقیقی از خطر بیماری در نواحی به منظور اختصاص بهتر منابع و ساخت اطلس بیماری‌ها. تاکنون مطالعات موردی متعددی انجام گرفته است که در آن از کابردهای مدل‌های نقشه‌بندی بیماری‌ها استفاده گردیده است. با این وجود جز در چند مطالعه اندک و به صورت محدود، روش‌های متعدد تحلیل نقشه‌های بیماری‌ها با هم مقایسه نشده و شرایط کلی آن‌ها مورد بررسی قرار نگرفته است. با توجه به اهمیت موضوع و کاربرد رو به گسترش نقشه‌بندی بیماری‌ها، در مطالعه حاضر مهم‌ترین مدل‌های نقشه‌بندی بیماری‌ها معرفی و مبانی تئوری، کاربردها، محاسن و معایب هر یک به اختصار بیان گردیده و از جنبه‌های مختلف مورد مقایسه قرار گرفته‌اند.

Title: ارتباط دریافت میوه و سبزی و سرطان كولوركتال: مروری بر شواهد موجود

http://hsr.mui.ac.ir/index.php/jhsr/article/view/1248

مقدمه: سرطان كولوركتال (CRC یا Colorectal Cancer) سومين سرطان شايع دنيا  و جزء 5 سرطان شایع در ایران می‌باشد. کاهش فعالیت‌های فیزیکی، تغییر رژیم غذایی افراد به سمت مصرف بیشتر غذاهای پر چرب و گوشت قرمز و مصرف کمتر غلات کامل، انواع میوه و سبزی باعث افزایش سرطان كولوركتال شده است. لذا هدف از این مطالعه مروری بر مطالعات موجود در زمینه مصرف میوه، سبزی و سرطان كولوركتال می‌باشد. روش‌ها: با استفاده از کلید واژه هایی مانند , Fruit, Vegetable Rectal cancer،  Colon Cancer، Colorectal cancer  cohort study, Randomized Trial در موتورهای جستجوي  Pub medو Google Scholar  مطالعات مرتبط انتخاب گردید، در نهایت60 مطالعه مورد بررسی قرار گرفت. یافته‌ها: بر اساس یافته‌های مطالعات اپیدمیولوژیک بین مصرف مرتب میوه و سبزی با کاهش سرطان كولوركتال ارتباط وجود دارد. نتایج مطالعات در مورد تأثیر میوه و سبزی بر روی پولیپ‌های آدنوماتوز متناقض است. در مطالعات کوهورت ارتباط ضعیف‌تری بین مصرف میوه، سبزی و خطر ابتلا به CRC نسبت به مطالعات مورد - شاهدی دیده شد. اجزا یا زیر گروه خاصی از میوه‌ها و سبزیجات با خطر ابتلا به  CRC ارتباط دارند. نتیجه‌گیری: افزایش مصرف میوه و سبزی باعث کاهش خطر سرطان کولورکتال نمی‌شود، ولی می‌توان گفت مصرف کم میوه و سبزی با افزایش خطر ابتلا به CRC ارتباط دارد. مصرف میوه‌ها و سبزیجات اثرات مطلوبی بر سلامت افراد و کاهش خطر سایر بیماری‌های مزمن دارند.

Title: بررسي انواع درد کمر ولگن در بارداری و ریسک فاکتورهای موثر بر ایجاد درد

http://hsr.mui.ac.ir/index.php/jhsr/article/view/1063

مقدمه: تقریبا 70 درصد زنان باردار به كمر دردهاي دوران بارداري مبتلا مي‌شوند. زنانی که پیش از بارداری نیز کمردرد داشته‌اند یا در بارداری قبلی کمردرد را تجربه کرده‌اند بیشتر در معرض ابتلا به کمردرد می‌باشند. هدف از انجام این مطالعه بررسی فراوانی انواع کمردرد در بارداری و ریسک فاکتورهای مؤثر بر آن می‌باشد. روش‌ها: این مطالعه توصیفی مقطعی به بررسی انواع دردکمر و لگن در 265 خانم باردار مراجعه‌کننده به مراکز بهداشتي درماني شهر اصفهان در سال 1391 پرداخت. خانم‌های مبتلا به دردکمر و لگن پس از معاینه و تشخیص، بر اساس نوع درد به سه گروه درد در ناحيه ساكروايلياك یا لگن (PGP یا pelvic girdle pain) ، درد در ناحيه تحتاني كمر (LBP یا low back pain) و درد در ناحيه فوقاني کمر (HBP یا high back pain) و بر اساس زمان شروع درد به چهار گروه 12-0 و 24-13 و 30-25 و 40-31 هفته طبقه‌بندی شدند. ابزار گردآوری داده‌ها پرسش‌نامه بود و روایی آن به‌صورت روایی محتوا بررسی و پایایی آن با استفاده از آزمون مجدد 95 درصد تعیین گردید. پرسش‌نامه‌ها از طریق مصاحبه تکمیل گردیدند. داده‌ها با استفاده از نرم‌افزارSPSS  نسخه 15 و آزمون‌های آماری کای دو ، آنالیز واریانس، کروسکال والیس و من ویتنی مورد تحلیل قرار گرفتند. در تمام آزمون‌ها سطح معنی‌داري05/0 P<مد نظر قرار گرفت. یافته‌ها: در بررسی انجام شده از 265 خانم باردار مبتلا به درد کمر و لگن شایع‌ترین نوع درد، درد پایین کمر ( 3/51 %) گزارش شد. پس از آن درد لگن با فراوانی 5/27 % ، درد ناحیه بالا و پایین کمر 8/6% و درد بالای کمر با فراوانی 6% دیده شدند. شایع‌ترین زمان بروز درد کمر یا لگن در سه ماهه اول بارداری (0-12 هفته) بود (6/42%). در سه ماهه اول حاملگی (هفته 12-0) بیشترین درد در ناحیه پایین کمر ( 1/53 %) ، در هفته‌های 24-13 در ناحیه تحتانی کمر (2/58%)، در هفته‌های 30-25 در ناحیه لگن (7/47 %) و در هفته‌های آخر بارداری 40-31 نیز در ناحیه لگن یا ساکروایلیاک (2/42 %) دیده شد. شایع‌ترین محل انتشار درد در خانم های مبتلا به درد پایین کمر ساق پا (6/31% ) ، در خانم‌های مبتلا به درد لگن ران‌ها ( 4/68% ) و در خانم های مبتلا به درد بالای کمر اکثریت، درد را بدون انتشار به محل دیگر بیان نمودند. عوامل مؤثر بر بروز درد کمر و لگن در بارداری سابقه تروما و سابقه درد کمر یا لگن در بارداری‌های قبلی بودند. بين درد لگن و كمر با ساير فاكتورها از جمله سن، BMI ، تعداد زایمان ، نوع زايمان، سن حاملگي، ، شغل و … رابطه معني‌دار يافت نشد (05/0 >P ). نتیجه‌گیری: ميزان شيوع درد کمر و لگن در زنان باردار ، بالا و بيشترين نوع درد نيز از نوع درد پایین کمر مي‌باشد و بر اين اساس مي‌توان نتيجه گرفت كه كمردرد در زنان باردار يك معضل بوده که براي رفع اين مشكل بايد چاره‌اي انديشيده شود. جهت كاهش آن، تشخیص به موقع و صحیح نوع درد وسپس توصیه به انجام حركات و تمرينات ورزشي مختص اين دوران زير نظر متخصص توان‌بخشي حایز اهمیت می‌باشد.

Title: بررسی میزان استفاده از مسواک و نخ دندان بر اساس سازه های الگوی اعتقاد بهداشتی توسط افراد مراجعه کننده به واحد درمانی دانشکده دنداتپزشکی دانشگاه علوم پزشکی اصفهان در سال1391

http://hsr.mui.ac.ir/index.php/jhsr/article/view/593

مقدمه: بیماری‌های دهان و دندان به عنوان شایع‌ترین بیماری‌ها در دنیا، با رفتارهاي فرد ارتباط تنگاتنك دارد. این مطالعه با هدف تعيين پيشگويي كنندگي سازه‌هاي الگوي اعتقاد بهداشتي در رفتارهای مرتبط با بهداشت دهان ودندان (مسواک زدن و استفاده از نخ دندان) در میانسالان صورت گرفت. روش‌ها: پژوهش حاضر يك مطالعه مقطعي توصیفی تحليلي بود كه روي 274 نفر از مراجعه‌كنندگان به دانشکده دندانپزشکی دانشگاه علوم پزشکی اصفهان که به شکل نمونه‌گیری تصادفی ساده انتخاب شده بودند، صورت گرفت. ابزار جمع آوري اطلاعات، پرسش‌نامه‌اي محقق ساخته بر اساس مدل اعتقاد بهداشتي بود. تجزيه و تحليل اطلاعات با استفاده از نرم افزار SPSS نسخه 20 و آزمون‌هاي آمار توصيفي و تحليلي صورت گرفت. سطح معني‌دار آزمون‌ها05/ 0در نظر گرفته شد. یافته‌ها: میانگین سنی شرکت‌کنندگان 47/11±45/30 سال بود. ارتباط معنی‌داری بین مشخصات دموگرافیک به جز خود ارزیابی وضعیت درآمد با رفتارهای بهداشتی سلامت دهان و دندان وجود نداشت. از بین سازه‌های مدل موانع درک شده مسواک و نخ دندان با (001/0P< ، 53/0- R=) بيشترين عامل پیش‌بين در رفتار بهداشتي دهان و دندان بود که به همراه منافع درک شده نخ دندان و منافع درک شده مسواک زدن38% رفتار استفاده از نخ دندان و 33% رفتار مسواک زدن را پیش‌بینی می‌کرد. نتیجه‌گیری: با طراحی و اجرای مداخلات آموزشی متمرکز برکاهش موانع، می‌توان افراد را به انجام رفتارهای مرتبط با بهداشت دهان و دندان تشویق نمود.

Title: كاربرد نانولوله‌های كربني تک‌جداره در جذب رنگ راکتیو قرمز 198 از محلول‌هاي آبي

http://hsr.mui.ac.ir/index.php/jhsr/article/view/1168

مقدمه: رنگ‌ها باعث مشکلات زیادی و یکی از منابع اصلی آلودگی محیط زیست هستند. بنابراین پساب‌های رنگی باید قبل از تخلیه، تصفیه شوند تا خطرات زیست‌محیطی کاهش يابد. یکی از پرکاربردترين روش‌های حذف رنگ از پساب، فرایند جذب است. نانولوله‌ها، جاذب‌های جدید با کاربردهای گسترده برای جذب انواع آلاینده‌ها می‌باشند. هدف از اين مطالعه بررسي امكان‌سنجي استفاده از نانولوله‌های كربني تک‌جداره به عنوان جاذب در فرآيند جذب رنگ راکتیو قرمز 198 از محلول‌هاي آبي می‌باشد. روش‌ها: اين مطالعه تجربي به صورت ناپيوسته انجام شد و اثر پارامترهاي مختلف زمان تماس، شدت اختلاط، غلظت اولیه رنگ، pH و دوز نانولوله‌هاي كربني تك‌جداره در حذف رنگ راکتیو قرمز 198بررسي شده است. یافته‌ها: نتایج نشان داد كه زمان مناسب جهت دستیابی به حداکثر جذب رنگ و حالت تعادل، 3 ساعت می‌باشد. با افزايش دوز جاذب از 1/0 به 9/0 گرم در ليتر راندمان حذف برای محلول رنگ راکتیو قرمز 198 با غلظت اولیه 25 میلی‌گرم در لیتر از 8/91% به 100% افزايش مي‌يابد. هم‌چنين مشخص گرديد که حداكثر ظرفيت جذب سطحي رنگ راکتیو قرمز 198 بر روي نانولوله‌هاي كربني تک‌جداره 98/7 ميلي‌گرم بر گرم در شرایط اسیدی (4=pH) اتفاق افتاده است. با كاهش غلظت اوليه رنگ راکتیو قرمز 198 از 50 به 25 ميلي‌گرم در ليتر در 4= pHو زمان 3 ساعت راندمان حذف از 9/96% به 100% افزايش يافته است.. نتیجه‌گیری: نتایج این مطالعه نشان داد که سینتیک واکنش با معادله درجه اول کاذب قابل توصیف بوده و ایزوترم جذب از هر دو مدل فروندلیچ و لانگموير تبعیت می‌نماید. به دليل اندازه كوچك، سطح مقطع زياد، شكل كريستالي و نظم شبكه‌اي منحصر به فرد و در نتيجه واكنش‌پذيري بسيار زياد و عملكرد قابل توجه نانولوله‌های كربني تک‌جداره به عنوان جاذب جهت حذف رنگ راکتیو قرمز 198 می تواند مؤثر باشد.

Title: تاثير رويکرد کوتاه مدت راه حل- محور بر کاهش تعارضات زناشويي شهر قم

http://hsr.mui.ac.ir/index.php/jhsr/article/view/1040

مقدمه: تعارض عبارت است از عدم توافق و مخالفت دو فرد با يكديگر، ناسازگاري نظرات و اهداف و رفتاري است كه در جهت مخالفت با ديگري صورت مي‌گيرد. هم‌چنين ستيزه‌اي بين افراد در اثر منافع ناهمسو و اختلاف اهداف و ادراكات مختلف است. تعارض‌های مخرب، مجموعه رخداد‌ها يا حوادثي هستند كه بر يك يا چند تن از اعضاي خانواده يا همه آن‌ها يا به طور كلي بر پيكره خانواده تحميل مي‌شوند و همه اعضاي خانواده را تحت تأثير قرار مي‌دهد. پژوهش حاضر به منظور بررسي اثر رويکرد راه حل - محور بر کاهش تعارضات زناشويي زوجین شهر قم صورت گرفته است. روش‌ها: اين پژوهش نيمه تجربي و از نوع پيش آزمون و پس آزمون با دو گروه آزمايش و گواه استفاده شده است. جامعه پژوهش شامل آن دسته از زوجين مراجعه‌کننده به مرکز مشاوره خانواده شهر قم مي‌باشد که به صورت خود معرف مراجعه کرده بودند. نمونه پژوهش شامل 50 زوج است که داوطلب داوطلبانه در مطالعه بودند، به صورت نمونه در دسترس انتخاب شدند و به صورت تصادفي در گروه آزمايش و کنترل قرار گرفتند. ابزار پژوهش شامل مشخصات دموگرافیک و پرسش‌نامه تعارضات زناشويي ثنايي بود. براي تجزيه و تحليل داده‌ها علاوه بر استفاده از روش‌هاي آمار توصيفي از روش تحليل کوواريانس استفاده شد. یافته‌ها: تحليل داده‌ها نشان داد رويکرد راه‌حل- محور بر تمام تعارض زوجي مؤثر است (089/24, F=001/0P<) و تمام زیر مقیاس‌های کاهش همکاري (4/7, F=001/0P<)، کاهش رابطه جنسي (4/50, F=001/0P<)، افزايش واکنش‌هاي هيجاني (89/10 ,F=001/0P<)، افزايش جلب حمايت فرزند (3/22, F=001/0P<)، افزايش رابطه با خويشاوندان خود (4/77, F=001/0P<)، کاهش رابطه با خويشاوندان و دوستان همسر (02/31, F=001/0P<)، جدا کردن امور مالي از يکديگر (1/71, F=001/0P<)، کاهش ارتباط مؤثر (73/1, F=001/0P<) مؤثر است. نتیجه‌گیری: ايجاد مهارت‌هاي حل مشکل در زوجين مي‌تواند از اختلافات بسياري جلوگيري کند. آموزش حل مسأله به عنوان يکي از مهارت‌هاي زندگي امروزه از اهميت خاصي برخوردار است. که در رويکرد راه حل- محور از اين فن زياد استفاده مي‌شود با استفاده از فنون مختلف اين رويکرد مي‌توان به زوجيني که به خاطر مشکلاتي که راه حل آن در دست خودشان است کمک کرد و رضايت از زندگي زناشويي شان را تقويت کرد.

Title: سرمايه اجتماعي دانشجويان دانشگاه علوم پزشكي تهران در سال 1390

http://hsr.mui.ac.ir/index.php/jhsr/article/view/840

چكيده مقدمه: سرمايه اجتماعي مجموعه هنجارهاي موجود در سيستم‌هاي اجتماعي است كه موجب ارتقاي سطح همكاري اعضاي آن جامعه گرديده و باعث پايين آمدن سطح هزينه‌هاي تبادلات و ارتباطات مي‌گردد. به دليل اهمیت سرمایه اجتماعی، اين پژوهش سعي دارد ميزان سرمايه اجتماعي و عوامل مؤثر بر آن را در دانشجويان دانشگاه علوم پژشكي تهران مورد بررسي قرار دهد. روش‌ها: : اين مطالعه، يك مطالعه‌ي مقطعي بود كه 400 نفر از دانشجويان دانشگاه علوم پزشكي تهران در آن شركت داشتند. شركت‌كنندگان، متناسب با جمعيت دانشجويي هر دانشكده به صورت تصادفی انتخاب شدند. ابزار گردآوري داده‌ها در اين مطالعه، پرسش‌نامه 36 سؤالي Bullen بود كه داراي هشت حيطه مي‌باشد. پس از جمع‌آوري داده‌ها، تجزيه و تحليل با استفاده از آزمون‌هاي  T-Testو ضريب همبستگي پيرسون و رگرسيون خطي در نرم‌افزار  SPSS.18انجام گرفت. یافته‌ها: ميانگين سرمايه اجتماعي در اين مطالعه، 12/44 مي‌باشد. كم‌ترين ميانگين، مربوط به حيطه مشاركت در اجتماعات محلي و بيشترين ميانگين، مربوط به حيطه‌ي ارتباط با دوستان و خانواده مي‌باشد. در اين مطالعه سرمايه اجتماعي با متغيرهاي محل سکونت و وضعیت اقتصادی ارتباط آماري معني‌داري داشت. نتیجه‌گیری: اين تحقيق نشان داد برخي حيطه‌هاي سرمايه اجتماعي با متغيرهايي مانند وضعيت اقتصادي، جنسيت، محل سكونت و رشته تحصيلي ارتباط دارد؛ مسؤولان مي‌توانند با در نظرگرفتن اين متغيرها و انجام مداخلات مناسب، ميزان سرمايه اجتماعي را در دانشجويان، افزايش دهند.

Title: مقایسه میزان اسیدهای آمینه ضروری موجود در انواع برنج مصرفی در شهر اصفهان در سال 1391

http://hsr.mui.ac.ir/index.php/jhsr/article/view/1213

مقدمه: پروتئین برای ساختمان بدن انسان و به عنوان یک منبع انرژی ضروری می‌باشد. کیفیت پروتئین بستگی به الگوی اسید آمینه آن دارد. از آنجایی که برنج بعد از نان قوت غالب مردم کشور ما است و برنج مصرفی در کشور در مناطق مختلف آب و هوایی کشت داده می‌شود و حتی نوع وارداتی آن نیز مورد مصرف می‌باشد، تعیین میزان اسیدهای آمینه ضروری انواع مختلف برنج جهت بررسی کیفیت پروتئین آنها لازم به نظر می‌رسد. بنابراین هدف از انجام این تحقیق مقایسه میزان اسیدهای آمینه ضروری موجود در انواع برنج تایلندی، شمالی، لنجانی و هندی درشهر اصفهان بود. روش‌ها: در این پژوهش تحلیلی، برای هر نوع برنج تعداد 11 نمونه و مجموعاً 44 نمونه به طور تصادفی از عمده فروشی‌های سطح شهر اصفهان خریداری شد. هر نمونه طبق روش استاندارد شماره 12/994 AOAC آماده‌سازی شده، با استفاده از دستگاه آمینواسید آنالیزور میزان انواع اسیدهای آمینه ضروری آن تعیین و محاسبه گردید. یافته‌ها: نتایج نشان داد که میانگین همه اسیدهای آمینه ضروری والین، ایزولوسین، لیزین، لوسین، تره اونین، متیونین، فنیل آلانین و هیستیدین در انواع برنج تایلندی، شمالی، هندی و لنجانی یکسان نمی‌باشد (001/0 P<). نتیجه‌گیری: نتایج نشان می دهد که کیفیت برنج لنجانی از لحاظ پروتئین و میزان اسیدهای آمینه ضروری برتر و برنج تایلندی ضعیف‌تر از سایر انواع برنج می‌باشد. این مطالعه می‌تواند گام کوچکی در جهت آغاز تدوین جدول ترکیبات غذایی جامع و کامل ایرانی می‌باشد.

Title: استفاده از امکانات جانبی فن آوری تلفن همراه در دانشجویان دانشگاه علوم پزشکی اصفهان

http://hsr.mui.ac.ir/index.php/jhsr/article/view/1180

مقدمه: بررسی رشد و گسترش تلفن همراه، حاکی از آن است که تاکنون هیچ فناوري در طول تاریخ بشر به شدت تلفن همراه فراگیر نشده است. از طرفی تلفن همراه، به مثابه عصاره فناوري‌هاي جهان روز، امکانات مختلفی را در خود گرد آورده است. مطالعه حاضر به استفاده از امکانات جانبی تلفن همراه در دانشجویان به عنوان مهم‌ترین و بزرگترین گروه استفاده کننده از خدمات و امکانات تلفن همراه پرداخته است. روش‌ها: اين پژوهش توصيفي- تحليلي مقطعي در 1300 نفر دانشجویان از دانشگاه علوم پزشکی اصفهان در سال 91-1390 انجام شد. اطلاعات با پرسش‌نامه دو قسمتی به روش خود ایفا جمع‌آوری شد. قسمت اول پرسش‌نامه شامل اطلاعات دموگرافیک شامل سن، جنس، وضعیت تأهل، مقطع تحصیلی و قسمت دوم شامل اطلاعات مربوط به امکانات جانبی تلفن همراه بود. تجزيه و تحليل داده‌ها با نرم‌افزارSPSS  نسخه 18 و روش‌هاي آماري توصيفي و استنباطي صورت گرفت. یافته‌ها: دختران به طور معنی‌داری بیش از پسران از سرویس پیام کوتاه و نرم‌افزارهای آموزشی نظیر لغت‌نامه استفاده می‌کردند در حالی که پسران بیش از دختران از تلفن همراه جهت ورود به اینترنت استفاده می‌کردند. میانگین کلی استفاده از امکانات جانبی تلفن همراه در پسران به طور معنی‌داری بالاتر از دختران بود همچنین پسران بیش از دختران گوشی تلفن همراه خود را تعویض کرده بودند (001/0 P≤). بر اساس نتایج تحلیل عاملی اکتشافی امکانات به پنج گروه استفاده چند رسانه‌ای، استفاده نرم‌افزاری، استفاده ابزاری، استفاده در جهت یادآوری، استفاده در جهت وقت شناسی تقسیم شدند که این پنج گروه بر روی هم درصد زیادی از واریانس داده‌ها (7/52%) را تبیین می‌کرد. نتیجه‌گیری: از میان قابلیت‌های تلفن همراه سرویس پیام کوتاه، ساعت زنگدار، آگاهی از ساعت، استفاده از نرم‌افزارهای آموزشی نظیر لغت‌نامه، دفترچه تلفن و حافظه جانبی بیشتر مورد استفاده دانشجویان قرار می‌گیرد و بیشترین میزان استفاده دانشجویان از گروه  قابلیت‌های چند رسانه‌ای یا دیداری شنیداری تلفن همراه است. می‌توان از تلفن همراه به عنوان ابزاری سودمند در جهت آموزش و اطلاع‌رسانی دانشجویان استفاده نمود.

Title: ارتباط سنجی درک از بیماری و آگاهی تغذیه‌ای با رفتار تغذیه‌ای در بیماران روستایی مبتلا به پرفشاری خون در شهرستان اردبیل

http://hsr.mui.ac.ir/index.php/jhsr/article/view/1254

مقدمه: آگاهی تغذیه‌ای و درک از بیماری از پرفشاری خون در رعایت رژیم غذایی به‌عنوان عاملی محوری برای کنترل پرفشاری خون اهمیت ویژه‌ای دارد این مطالعه با هدف تعیین ارتباط ادراکات بیماری و آگاهی تغذیه‌ای با رفتارهای تغذیه‌ای در بیماران روستایی مبتلا به پرفشاری خون انجام شد. روش‌ها: در یک مطالعه توصیفی تحلیلی تعداد 671 نفر از بیماران پرفشاری خون روستایی شهرستان اردبیل در سال 1392 به روش نمونه‌گیری چند مرحله‌ای تصادفی ساده با رعایت معیارهای ورود انتخاب شدند. ابزار جمع‌آوری اطلاعات پرسش‌نامه 4 قسمتی شامل اطلاعات دموگرافیک، سؤالات آگاهی تغذیه‌ای، سؤالات ادراک از بیماری و پرسش‌نامه ثبت 3 روزه مواد غذایی بود. داده‌ها با مصاحبه حضوری توسط بهورز آموزش دیده جمع‌آوری و اطلاعات تغذیه‌ای با نرم افزار Nutritionist 4 (N4) شامل درشت مغذی‌ها (مقدار چربی، کلسترول، پروتئین، کربوهیدرات و انرژی کل دریافتی) استخراج و با استفاده از نرم‌افزارهای Excel 2007 , SPSS – 18 و آزمون‌های همبستگی پیرسون، رگرسیون خطی چندگانه و تی مستقل با در نظر گرفتن سطح معنی‌داری 05/0 مورد تجزیه تحلیل قرار گرفت. یافته‌ها: 74% از شرکت‌کنندگان از جنس زن بودند، میانگین و انحراف معیار سن بیماران 4/6 ± 2/50 سال، مدت ابتلا به این بیماری 0/4 ± 9/5 سال و اکثریت افراد  9/75% (509 نفر) دارای تحصیلات ابتدایی بودند. بیماران از ادراک و آگاهی متوسطی برخوردار بودند. مقدار چربی دریافتی 1/92 گرم، پروتئین 3/110 گرم و کربوهیدرات 317 گرم در روز و مقدار انرژی دریافتی روزانه به‌طور میانگین 2539 کیلوکالری به‌دست آمد. میانگین نمره ادراک با آگاهی تغذیه‌ای ارتباط مستقیم معنی‌دار (583/0=r و 01/0>P) و ارتباط معکوس معنی‌داری با میانگین چربی(352/0- =r و 01/0>P) ، کلسترول(09/0- =r و 05/0>P) ، انرژی دریافتی(318/0- =r و 01/0>P) ، فشار خون سیستول (614/0- =r و 01/0>P) و دیاستول (400/0- =r و 01/0>P) داشت. هم‌چنین آگاهی تغذیه‌ای نیز ارتباط معکوس معنی‌داری با میانگین چربی، کلسترول، انرژی دریافتی، فشارخون سیستول و دیاستول داشت (05/0>P). مدل رگرسیونی نشان داد که آگاهی تغذیه‌ای و ادراک توان پیشگویی 8/47% تغییرات فشارخون سیستولی را داشتند. نتیجه‌گیری: بیماران مورد مطالعه از ادراکات تغذیه‌ای نامناسبی برخوردار بودند هم‌چنین چربی دریافتی و انرژی دریافتی بیماران بیش از مقادیر توصیه شده توسط سازمان‌های معتبر علمی است.

Title: بررسی کیفیت میکروبی آب شرب مصرفی اتوبوس‌های بیرون شهری پایانه شهید کاراندیش شیراز

http://hsr.mui.ac.ir/index.php/jhsr/article/view/1291

مقدمه: در ایران به دلیل فقدان سیستم حمل و نقل هوایی و ریلی منظم و در دسترس، بیشترین میزان مسافرت‌ها با استفاده از اتوبوس صورت می‌گیرد و اكثرا از آب اتوبوس‌ها جهت شرب مسافران استفاده می‌گردد كه در صورت آلوده بودن خطر جدی جهت اشاعه بیماری‌های ناشی از آشاميدن آب آلوده محسوب می‌گردد. مطالعه حاضر به منظور بررسی کیفیت میکروبی آب در اتوبوس‌های برون شهری ترمینال کاراندیش شیراز انجام شد. روش‌ها: در اين مطالعه، 48 نمونه به روش سهمیه‌ای انتخاب شد و آلودگی میکروبی آب (کل کلیفرم و کلیفرم گوارشی) با استفاده از آزمایش MPN به روش تخمیر 9 لوله‌ای مورد بررسی قرار گرفت. میزان کلر باقی مانده، کدورت،pH  و دما سنجش شد و داده ها با استفاده از نرم افزار SPSS نسخه 18 آناليز گرديد. یافته‌ها: نتایج نشان داد که در 75/18 درصد موارد کل کلیفرم و در 17/4 درصد موارد کلیفرم مدفوعی مثبت بود. میزان کدورت فقط در 3/8 درصد موارد در حد استاندارد کیفی آب آشامیدنی بود و5/62 درصد از مخازن آب اتوبوس‌ها فاقد كلر بودند. نتیجه‌گیری: نتیجه‌گیری: مقايسه نتايج حاصل از اين تحقيق با مطالعات مشابه انجام شده نشان داد که آلودگی مدفوعی در آب آبخوری‌های اتوبوس‌های پایانه شهید کاراندیش شیراز ( 17/4%) نسبت به کرمانشاه، بندر عباس و کرمان کمتر بوده ولی این آلودگی در آب آبخوری‌های اتوبوس‌های شیراز نسبت به اصفهان بیشتر بوده است.

Title: بررسی فراوانی نارضایتی زناشویی زوجین دارای همسران مبتلا به سندرم قبل از قاعدگی در شهر یزد

http://hsr.mui.ac.ir/index.php/jhsr/article/view/1298

مقدمه: رضایت زناشویی از احساسات عینی از خوشنودی، رضایت، و لذت تجربه شده زن و شوهر تعریف می شود. سندرم پیش از قاعدگی سبب اختلال در روابط و فعالیت‌های روزانه می‌گردد. هدف این مطالعه تعیین فراوانی نارضایتی زناشویی زوجین دارای همسران مبتلا به سندرم قبل از قاعدگی در شهر یزد بود. روش‌ها: حجم نمونه در این پژوهش توصیفی تحلیلی 240 زوج جوان مراجعه کننده به مراکز بهداشتی درمانی شهر یزد بود. ابزار مورد استفاده پرسش‌نامه استاندارد غربالگری علایم قبل از قاعدگی (PSST یا Premenstrual Symptoms Screening Tool) ، پرسش‌نامه استاندارد (IMS یا Index of Marital Satisfaction) برای تعیین رضایت زناشویی زوجین و پرسش‌نامه محقق ساخته برای سنجش میزان آگاهی و عملکرد مردان درخصوص سندرم قبل از قاعدگی بود. روایی و پایایی پرسش‌نامه بعد از مطالعه مقدماتی تأیید گردید. اطلاعات توسط نرم افزار آماري 18 SPSSو با استفاده از آزمون‌های آماری T-Test، ANOVA، ضریب هم بستگی، 2χ و نيز جداول توزيع فراواني تجزیه و تحلیل شد. یافته‌ها: یافته‌ها بیانگر این بودکه نارضایتی مردان 1/72% و نارضایتی زنان 7/56% و رابطه بین نمره رضایت زناشویی مرد و زن معنی‌دار بود (001/0> p). همبستگی بین نمره آگاهی مرد با نارضایتی زن و نارضایتی مرد معنی‌دار نبود. ولی رابطه معنی‌دار معکوسی بین نمره عملکرد مردان در مورد سندرم قبل از قاعدگی با نارضایتی زن (001/0> p) و نارضایتی مرد (001/0> p) وجود داشت. نتیجه‌گیری: گرچه  ارتباط معنی‌دار آماری بین آگاهی مردان از سندرم قبل از قاعدگی و رضایت زناشویی دیده نشد لیکن عملکرد آن‌ها در مورد سندرم قبل از قاعدگی بر رضایت زناشویی تأثیرگذار می‌باشد. با توجه به ارتباط بین نمره آگاهی و عملکرد، می‌توان انتظار داشت که با افزایش آگاهی مردان در زمینه سندرم قبل از قاعدگی می‌توان رضایت زناشویی را تغییر داد.

Title: بررسي قابليت استفاده از پساب تصفيه خانه‌هاي فاضلاب شهر اصفهان در آبیاری محدود

http://hsr.mui.ac.ir/index.php/jhsr/article/view/1474

مقدمه: امروزه در بسياري از کشورها پساب بخش مهمي از منابع آب تجديد شونده را شامل مي‌شود. در اين خصوص توجه به کيفيت پساب مورد استفاده و رعايت استانداردهاي تدوين شده مهم است. هدف از انجام این مطالعه، بررسي قابليت استفاده از پساب تصفيه‌خانه‌هاي فاضلاب شهر اصفهان در آبیاری محدود می‌باشد. روش‌ها: این مطالعه از نوع توصیفی- تحلیلی است که برای تعيين امکان استفاده از پساب تصفيه‌خانه‌هاي شمال و جنوب شهر اصفهان، نمونه‌های پساب از نظر پارامترهای BOD5، COD، TSS،PH،SAR، قلیائیت ، کلسيم، منيزيم، سديم، هدايت الکتريکي، بور، نیترات، سولفات، فسفات و کلرايد مورد آناليز قرار گرفت. یافته‌ها: میانگین پارامترهای BOD5 و COD در هر دو تصفیه‌خانه مورد مطالعه در حد استانداردهای محیط زیست ایران بوده است ولی استانداردهای EPA را تأمین نمی‌کند. مقادیر PH پساب خروجی هر دو تصفیه‌خانه در محدوده مجاز (9-6) اندازه‌گیری شد. غلظت بور و نسبت جذب سدیم در پساب تصفیه‌خانه شمال برای آبیاری، در حد خوب، درصد سدیم در حد مجاز و غلظت کلراید و EC نامناسب ارزیابی شد. در پساب تصفیه‌خانه جنوب، بور و SAR در حد عالی، غلظت کلراید و EC در حد مجاز و درصد سدیم در حد خوب اندازه‌گیری شد. نتیجه‌گیری: بر اساس آزمون آماری t مستقل، تفاوت معنی‌داری بین کیفیت پساب خروجی تصفیه خانه‌های شمال و جنوب اصفهان مشاهده شد (05/0P<). تفاوت معنی‌داری بین کیفیت پساب خروجی و مقادیر استاندارد مشاهده نشد (05/0P<). با توجه بیشتر به بهره‌برداری واحدها و به خصوص بهره‌برداری از یک فرایند مناسب گندزدایی، پسابی با کیفیت مناسب برای مصارف کشاورزی تامین می‌شود.

Title: کاربرد الگوی تصورات و تمایلات در توصیف رفتار سوء مصرف ریتالین در بین دانشجویان

http://hsr.mui.ac.ir/index.php/jhsr/article/view/1510

مقدمه: ریتالین از مشتقات گروه آمفتامین‌ها می‌باشد که امکان سوء مصرف آن به خصوص در بین دانشجویان وجود دارد. مطالعه حاضر با هدف تعیین نگرش، نرم‌های انتزاعی، تصورات خطر، تمایلات و قصد رفتاری سوء مصرف ریتالین در بین دانشجویان انجام گرفت. روش‌ها: این مطالعه از نوع توصیفی مقطعي بود و در میان 264 نفر از دانشجویان دانشگاه علوم پزشکی اصفهان در بهار سال 1392 انجام شد. شرکت‌کنندگان به روش نمونه‌گيري تصادفی ساده و با احتمال متناسب با حجم انتخاب شده و اطلاعات به وسیله پرسش‌نامه، به صورت خودگزارش دهی جمع‌آوری گردید. داده‌ها با استفاده از نرم افزار آماری SPSS ویرایش 21 مورد توصیف قرار گرفت. یافته‌ها: میانگین سن دانشجویان 28/2±02/23 سال بود. 6/6 درصد از دانشجویان تجربه سوء مصرف ریتالین را گزارش کردند. شرکت‌کنندگان به ترتیب 7/47، 6/48، 48، 5/43 و 6/52 درصد حداكثر نمره قابل اكتساب برای حیطه‌های نگرش، نرم‌های انتزاعی، تصورات خطر، تمایلات رفتاری و قصد رفتاری مصرف ریتالین را دریافت کردند. نتیجه‌گیری: یافته‌ها نشان‌دهنده قصد بیشتر مصرف ریتالین در ایام امتحانات بود، به نظر می رسد آموزش مهارت‌های مطالعه به دانشجویان در پیشگیری از سوء مصرف ریتالین نتایج سودمندی را به دنبال داشته باشد.

Title: استفاده از كيسه آب گرم در تسكين درد بيماران مبتلا به كمردرد حاد

http://hsr.mui.ac.ir/index.php/jhsr/article/view/1608

مقدمه: کمر درد حاد يکي از شايع‌ترين بيماري‌ها مي‌باشد كه بيش از 90٪ بزرگسالان حداقل يک بار در طول زندگي آن را تجربه مي‌كنند. مطالعه حاضر با هدف تعیین اثربخشی استفاده از كيسه آب گرم در تسكين درد بيماران مبتلا به كمردرد حاد انجام گرفت. روش‌ها: این مطالعه کارآزمایی بالینی بر روی بیماران رنج سني 20 تا 50 سال مراجعه کننده به کلینیک ارتوپدی بیمارستان آیت اله کاشانی شهرکرد در سال 1391 انجام گرفت. در این مطالعه 60 نفر بیمار مبتلا به کمردرد حاد به روش نمونه‌گيري آسان انتخاب شدند که از این تعداد 2 نفر به دلیل انصراف از ادامه درمان از مطالعه ما خارج شدند. در نهايت مداخله بر روی 58 بیمار كه پس از همسان‌سازي به طور تصادفی در دو گروه (مداخله تحت درمان با کیسه آب گرم به همراه درمان روتين (ناپروکسن) و گروه كنترل تنها تحت درمان روتين (ناپروكسن)) قرار گرفتند، انجام شد. کليه بيماران در روزهاي اول، سوم، هشتم و پانزدهم معاينه و درد بيماران بر اساس پرسش‌نامه درد مک گیل ارزيابي و ثبت گرديد. داده‌ها توسط نرم افزار17 SPSS و آزمون‌های آماری توصیفی، تي‌زوج، آناليز واريانس و مجذور كاي دو در سطح معني‌داري 05/0P< مورد تجزیه و تحلیل قرار گرفتند. يافته‌ها: در این مطالعه 52٪ بیماران زن و 48٪ مرد با میانگین سنی 34 سال بودند. يافته‌ها نشان داد شدت درد در معاينه روز 15در مقايسه با روزهاي 7 و 3 در گروه تحت درمان با كيسه آب گرم به طور معنی‌داری كمتر از گروه کنترل بود (02/0≥P). ميانگين نمره درد كلي در معاينه روز اول و روز پانزدهم در گروه مداخله 70/12 و 75/0و در گروه كنترل96/12 و 58/5 بود. نتیجه‌گیری: نتایج به دست آمده نشان می‌دهند که استفاده مداوم از كيسه آب گرم به همراه داروهاي مسكن در مقايسه با مصرف مسكن‌ها به تنهايي (كنترل) خيلي بيشتر می‌تواند موجب تسكين درد در بیمارانی که از کمردرد حاد رنج می‌برند، شود.

Title: ارزیابی کارآیی فرایند الکتروشیمیایی در حذف کروم شش ظرفیتی از فاضلاب مصنوعی با استفاده از الکترودهای آهن و کربن

http://hsr.mui.ac.ir/index.php/jhsr/article/view/1370

چكيده   مقدمه: فلزات سنگین موجود در فاضلاب‌های صنعتی از جمله کروم (VI)، از آلاينده‌هاي اصلي براي محيط زيست به شمار مي رود. این تحقیق با هدف ارزیابی کارایی فرآیند الکتروشیمیایی با مقایسه عملکرد دو الکترود آهن و کربن جهت حذف کروم (VI) از پساب مصنوعی انجام گرفت. روش‌ها: یک سلول از جنس پلکسی گلاس با حجم 95/1 لیتر حاوی دو نوع الکترود آهن و گرافیت هادی جریان برق هر کدام با ابعاد 1/0×3×10 سانتی‌متر (ضخامت×عرض×ارتفاع) طراحی و ساخته شد ومورد استفاده قرار گرفت. آزمایش‌ها در شرایط مختلف pH، غلظت‌های ورودی کروم شش ظرفیتی، مقادیر مختلف الکترولیت و شدت متنوعی از جریان الکتریسیته، در زمان‌های ماند مشخص انجام گردید. سنجش کروم (VI) نمونه‌ها با استفاده از روش استاندارد دی فنیل هیدرازین انجام گرفت. یافته‌ها: راندمان حذف حداکثر با الکترود آهن و کربن به‌ترتیب در محدوده 8/99% و 3/88% مشاهده گردید. کمترین میزان کروم Cr+3 خروجی برای الکترود آهن و در حدود mg/l 05/0 ثبت گردید. نتیجه‌گیری: نتایج حاصله نشان داد که در بهره برداری از یک سلول الکتروشیمیایی، راندمان حذف کروم شش ظرفیتی بوسیله الکترود آهن در شرایط فرآیندی یکسان بیشتر از الکترود کربن است. به‌طور کلی این روش علی‌رغم مصرف زیاد انرژی الکتریکی، راندمان قابل قبولی در حذف کروم شش ظرفیتی از پساب‌های دست‌ساز در مقیاس آزمایشگاهی دارد و می تواند یکی از روش های بالقوه در حذف فلزات سنگین در مقیاس صنعتی باشد.

Title: بررسی پیامدهای اجتماعی نازایی بر خانواده در شهر اصفهان

http://hsr.mui.ac.ir/index.php/jhsr/article/view/1741

مقدمه: عوارض مخرب نازایی علاوه بر روابط بین زوج‌ها بر سایر جنبه‌های زندگی آنها تأثیر می‌گذارد و عملکرد فردی، اجتماعی و حرفه‌ای آنها را مختل می‌کند. هدف پژوهش حاضر مطالعه پیامدهای اجتماعی نازایی بر خانواده در شهر اصفهان است. روش‌ها: تحقیق حاضر از نوع پیمایشی و ابزار جمع‌آوری اطلاعات پرسش‌نامه است. این مطالعه به صورت مقطعی بر روی 384 زن نابارور مراجعه کننده به مرکز باروری و ناباروری اصفهان به شیوه نمونه‌گیری در دسترس انجام شد. برای سنجش اعتبار تحقیق از اعتبار صوری و برای سنجش پایایی از ضریب آلفای کرونباخ استفاده شده است. اطلاعات حاصل با آزمون کای اسکور، اسپیرمن و پیرسون مورد ارزیابی قرار گرفت. یافته‌ها: نتایج تحقیق نشان داد بین طول مدت نازایی و سلامت روحی و روانی (05/0P< , 896/152=χ)، تعارضات زناشویی (05/0P< , 322/21   2=χ)، احتمال طلاق (05/0P< , 831/38 2=χ)، و کاهش روابط اجتماعی (05/0P< , 857/262=χ )، رابطه معنی‌داری وجود دارد. همچنین بین سلامت روحی و روانی زنان نابارور و تحصیلات (05/0P< , 151/0-  r=) و دینداری (05/0P< , 345/18 2=χ) آنها رابطه معنی‌داری وجود دارد. از سوی دیگر یافته‌های پژوهش نشان داد بین سلامت روحی و روانی زنان نابارور و سن (05/0P>. , 014/0 r=) ، اشتغال (05/0P> , 025/0r= ) و محل سکونت (05/0P> , 065/0r=) آنها رابطه معنی‌داری وجود ندارد. نتیجه‌گیری: نتایج تحقیق نشان داد که زنان نابارور در مقایسه با زنان بارور به میزان قابل توجهی از ناراحتی‌های روحی- روانی رنج می‌برند و این مسأله می‌تواند بر جنبه‌های دیگر زندگی آنها از جمله روابط اجتماعی و اختلافات خانوادگی آنها تاثیر منفی بگذارد.

Title: بررسی تراکم بیوآئروسل‌ها در هوای محیط‌های مختلف داخل ساختمان

http://hsr.mui.ac.ir/index.php/jhsr/article/view/1591

مقدمه: بيوآئروسل‌ها به ذرات بیولوژیک منتقله توسط هوا گفته مي‎شود كه شامل ارگانيسم‌هاي زنده مانند باكتري‌ها، قارچ‎ها و متابوليت‎هاي ناشي از آن‌ها نظير اندوتوكسين‎ها مي‎باشند. اثرات ناشي از بيوآئروسل‌ها مانند بيماري‌هاي عفوني و تنفسي، اثرات سمي حاد، آلرژي و سرطان طي چند سال اخير مورد توجه قرار گرفته است. هدف از این تحقیق بررسی میزان تراکم بیوآئروسل‌ها شامل باکتری و قارچ در محیط‌های داخلی (مسکونی، اداری و آموزشی) می‌باشد. روش‌ها: این مطالعه توصیفی- مقطعی در سال 1391به منظور بررسی میزان باکتری‌ها و قارچ‌ها در هوای محیط‌های داخلی شامل: محیط‌های مسکونی، اداری و آموزشی با استفاده از نمونه‌بردار تک مرحله‌ای آندرسون انجام شد و در مجموع 240 نمونه (60 نمونه باکتری و 60 نمونه قارچ با تکرار) برداشت شد. اثر عوامل محیطی مانند دما و رطوبت نیز بر میزان تراکم بیوآئروسل‌ها مورد بررسی قرار گرفت. یافته‌ها: هوای محیط‌های مسکونی با میانگین تراکم باکتری CFU/m3 944 بالاترین غلظت باکتری را نسبت به سایر محیط‌های داخلی داشت. هم‌چنین کلاس مدرسه با میانگین تراکم قارچ  CFU/m3 102 دارای بیشترین بار آلودگی قارچی بود. از طرفی کمترین تراکم باکتری و قارچ با میانگین کلی CFU/m3 132 و CFU/m3 36 به ترتیب مربوط به محیط‌های اداری و کلاس دانشگاهی بوده است نتیجه‌گیری: با توجه به نتایج حاصله، افراد در محیط‌های داخلی به‌ویژه فضاهای مسکونی در معرض غلظت‌های بالایی از بیوآئروسل‌ها قرار دارند و تراکم باکتری‌های هوابرد و قارچ در هوای فضاهای بسته مورد بررسی بیش از مقادیر پیشنهادی ارایه شده توسط WHO (  CFU/m3500) می‌باشد، که می‌تواند باعث ایجاد خطرات بهداشتی و بیماری‌های تنفسی به ویژه در کودکان و افراد حساس شود.

Title: بررسی تأثیر مداخله آموزشی تركيبي مبتنی برمدل اعتقاد بهداشتی دراتخاذ رفتارهای پیشگیری‌کننده از آلزایمر در سالمندان تحت پوشش مراکز بهداشتی درمانی شهرستان اردبیل

http://hsr.mui.ac.ir/index.php/jhsr/article/view/1457

مقدمه: توسعه رفتارهای بهداشتی جهت پیشگیری از آلزایمر در افرادی که در معرض خطر ابتلا به این بیماری هستند بسیار حایز اهمیت می باشد. مطالعه حاضر با هدف تعيين تأثیر آموزش بر اساس الگوی اعتقاد بهداشتی دراتخاذ رفتارهای پیشگیری‌کننده از آلزایمر انجام شد. روش‌ها: پژوهش حاضریک مطالعه نیمه تجربی است که در آن 148 نفر از سالمندان تحت پوشش مراکز بهداشتی درمانی شهر اردبیل به صورت تخصیص تصادفی به دو گروه مداخله ومقایسه تقسیم شدند. قبل از اجرای برنامه آموزشی، هر دو گروه پرسش‌نامه محقق ساخته را که روایی وپایایی آن به تأیید رسید تکمیل کردند،سپس افرادگروه آزمون،آموزش‌های لازم را در مورد بیماری آلزایمر و راه‌های پیشگیری از آن به صورت ترکیبی شامل سخنرانی ، بحث گروهی ، پخش کلیپ آموزشی وپرسش وپاسخ در سه جلسه (طی 3 ماه یادآور تلفنی) دریافت نمودند و دو گروه 3 ماه بعد مجددا پرسش‌نامه را تکمیل کردند. در نهایت اطلاعات جمع‌آوری شده با استفاده از نرم‌افزار 16 SPSSمورد تجزیه وتحلیل قرار گرفت. سطح معنی‌داری در این مطالعه کمتر از 05/0 در نظر گرفته شد. یافته‌ها: میانگین سنی گروه مورد مطالعه درگروه مداخله 9/7 ± 9/68 سال و در گروه مقایسه 6/7±8/68 سال بود. قبل از مداخله بین میانگین نمره حساسیت درک شده‌، شدت درک شده ، منافع و موانع درک شده، راهنمای عمل وعملکرد در دو گروه قبل از مداخله آموزشی اختلاف معنی‌داری وجود نداشت (05/0<p)، اما بعد از مداخله ، این اختلاف معنی‌دار بود (001/0P<). درگروه مداخله میانگین نمرات آگاهی و اجزای مدل بعد از مداخله آموزشی افزایش معنی‌داری یافت (001/0P <). هم‌چنین میانگین نمره عملکرد نیز در گروه مداخله 3 ماه بعد از آموزش افزایش معنی‌داری داشت (001/0 P<). نتیجه‌گیری: یافته‌های این بررسی کارایی مدل اعتقاد بهداشتی را در اتخاذ رفتارهای پیشگیری‌کننده از آلزایمر در سالمندان نشان داد. لذا پیشنهاد می‌شود در مراکز بهداشتی درمانی از مداخله آموزشی مبتنی بر این مدل بهره گرفته شود.

Title: ارزیابی آسیب پذیری آبخوان واقع در مکان جدید دفن پسماند شهری اصفهان بر اساس مدل دراستیک

http://hsr.mui.ac.ir/index.php/jhsr/article/view/1260

مقدمه: شیرابه حاصل از مکان دفن پسماند جامد باعث آلودگی آب‌های زیرزمینی می‌شود. ارزیابی آسیب‌پذیری آلودگی آب‌های زیرزمینی ابزاری توانمند جهت توصیف حفاظت از این چنین مناطقی است که متأثر از آلاینده‌ها هستند. هدف اصلی این تحقیق بررسی آسیب‌پذیری آلودگی آبخوان واقع در مکان دفن جدید زباله‌های شهر اصفهان به شیرابه با استفاده از روش دراستیک در محیط سامانه اطلاعات جغرافیایی GIS است. روش‌ها: جهت انجام این تحقیق هفت پارامتر هیدروژئولوژیکی تشکیل‌دهنده مدل دراستیک شامل عمق آب، تغذیه خالص، محیط آبخوان، محیط خاک، توپوگرافی، اثرات ناحیه غیر اشباع و هدایت هیدرولیکی مورد استفاده قرار گرفت. از نرم‌افزارArcGIS 9.3 جهت تهیه نقشه‌های آسیب‌پذیری از طریق همپوشانی لایه‌های هیدروژئولوژیکی استفاده شده است. یافته‌ها: نتایج مطالعه نشان می‌دهد که نمره به دست آمده از مدل دراستیک 115، 124 و 130 می‌باشد و پتانسیل آلودگی آبخوان به آلودگی‌های حاصل از شیرابه مکان دفن پسماند شهری متوسط می‌باشد. نتیجه‌گیری: استفاده از مدل دراستیک یک روش ارزشمند برای بررسی و ارزیابی پتانسیل آلودگی آب‌های زیرزمینی توسط شیرابه حاصل از مکان دفن پسماند است. GIS به عنوان ابزاری سودمند برای مسؤولین محلی که مسؤولیت مدیریت دفن پسماند شهری را بر عهده دارند قابل کاربرد است.
